# Three weekly versus weekly concurrent cisplatin: safety propensity score analysis on 166 head and neck cancer patients

**DOI:** 10.1186/s13014-021-01966-4

**Published:** 2021-12-20

**Authors:** Michela Buglione, Daniela Alterio, Marta Maddalo, Diana Greco, Marianna Alessandra Gerardi, Davide Tomasini, Ludovica Pegurri, Matteo Augugliaro, Giulia Marvaso, Irene Turturici, Andrea Guerini, Mohssen Ansarin, Luigi Spiazzi, Loredana Costa, Maria Cossu Rocca, Stefano Maria Magrini, Barbara Alicja Jereczek-Fossa

**Affiliations:** 1 Radiation Oncology Department, University and ASST Spedali Civili, Brescia, Italy; 2 Division of Radiotherapy, Radiation Oncology Department, IEO European Institute of Oncology, IRCCS, Via Ripamonti 435, 20141 Milan, Italy; 3 Department of Oncology and Hemato-Oncology, University of Milan, Milan, Italy; 4 Division of Head and Neck Surgery, IEO European Institute of Oncology, IRCCS, Milan, Italy; 5 Medical Physics, ASST Spedali Civili, Brescia, Italy; 6 Division of Medical Oncology, IEO European Institute of Oncology, IRCCS, Milan, Italy

**Keywords:** H&N cancer, Radiotherapy, Chemotherapy, Weekly-CDDP, 3weekly-CDDP

## Abstract

**Background:**

Radio-chemotherapy with CDDP is the standard for H&N squamous cell cancer. CDDP 100 mg/m^2^/q3 is the standard; alternative schedules are used to reduce toxicity, mostly 40 mg/m^2^/q1.

**Methods:**

Patients were treated from 1/2010 to 1/2017 in two Radiation Oncology Centres. Propensity score analysis (PS) was retrospectively used to compare these two schedules.

**Results:**

Patients analyzed were 166. Most (114/166) had 1w-CDDP while 52 had 3w-CDDP. In the 3w-CDDP group, patients were younger, with better performance status, smaller disease extent and a more common nodal involvement than in the 1w-CDDP. Acute toxicity was similar in the groups. Treatment compliance was lower in the w-CCDP. Overall survival before PS was better for female, for oropharyngeal disease and for 3w-CDDP group. After PS, survival was not related to the CDDP schedule.

**Conclusions:**

3w-CDDP remains the standard for fit patients, weekly schedule could be safely used in selected patients.

## Introduction

For decades, CDDP has been used in the management of locally advanced squamous cell carcinoma of the head and neck (LAHNSCC) in order to enhance the tumoricidal activity of radiation. Among the various CDDP schedules proposed, differing in frequency, dose, and administration, there is level 1 evidence for improvement in loco-regional control and/or overall survival, achieved by three-weekly high-dose (100 mg/m^2^) cisplatin concurrently with conventional external beam radiotherapy, when compared with radiotherapy alone. The supporting data originate from four large randomized phase III trials investigating the role of cisplatin in both the definitive and postoperative settings [1–4].

Since three-weekly cisplatin (3w-CDDP) causes significant acute toxicity in more than three quarters of patients, many patients are likely to receive sub-optimal cumulative cisplatin dose and dose intensity. This could hamper treatment outcomes and require a proper patient’s selection.

Low-dose weekly cisplatin (1w-CDDP) regimes have gained large clinical acceptance, replacing the standard 3w-CDDP schedule at many institutions in daily clinical practice. The background of this choice is the assumption that low-dose, 1w-CDDP could increases treatment compliance maintaining dose intensity and avoiding interruptions of radiotherapy [5]. It could also reduce chemotherapy-related acute and late side effects, facilitate dose adjustments according to clinical conditions during the treatment and therefore outpatient management, with lower hospitalization rates. Several retrospective and prospective studies [6–9], as well as different systematic reviews and meta-analysis [10–13] comparing 1w- and 3w- schedules, showed conflicting and inconclusive results, both in terms of survival outcomes and toxicity profile. Indeed, while most of these studies confirmed that oncologic outcome seems to be similar between the two regimens, hematologic toxicity showed not homogeneous findings among the different analyzed cohort.

Different prospective randomized trial are actually ongoing in curative setting of both LAHNSCC and nasopharyngeal cancer (NCT03998696, NCT03649048, NCT01171781, JPRN-jRCTs031180135, NCT03998696, NCT03649048). Weekly 1w-schedules have also been included in de-intensification trials for human papilloma virus-related tumors (NCT01530997, NCT01687413).

Therefore, waiting for definitive results, there is an unmet need to provide literature data on homogenous cohorts of patients treated with 1w-CDDP to guide the daily clinical practice.

In this contest, the main objective of this retrospective analysis is to compare, in a real-life setting of patients treated with definitive chemo-radiotherapy, two chemotherapy schedules (1w-CDDP 40 mg/m^2^ vs 3w-CDDP 100 mg/m^2^) concomitant to radical radiotherapy in locally advanced head and neck cancers, in terms of acute and overall and relapse free survival. The Propensity Score matched analysis should help to reduce the selection biases that are usually present in a retrospective series.

## Materials and methods

Patients enrolled in this retrospective analysis have been treated between January 1st, 2010 and January 30th, 2017 for LAHNSCC (oropharynx, hypopharynx and larynx) at the Radiation Oncology Departments of the Brescia University (“O. Alberti”, ASST-Spedali Civili—IRA) and of the European Institute of Oncology (IEO IRCCS)/University of Milan, Italy.

All patients had concomitant CDDP-based definitive chemo-radiotherapy. Two different CDDP schedules were used in the two Institutions: 100 mg/m^2^ every three weeks (3w-CDDP, IEO) and weekly 40 mg/m^2^ (1w-CDDP, IRA). Patients treated with adjuvant chemo-radiotherapy were not included in the analysis. In order to reduce the variability related to the patient’s body surface differences, the dose was considered as dose/m^2^ (ratio of total CDDP dose received by each patient and his/her body surface).

Data were retrospectively collected using a database where all the clinical and therapeutic features were entered.

The ethical committee of the two Institutions approved/notified the study.

Stage classification was carried out in accordance with the TNM classification system, VII Ed. [14].

Acute radiation and chemotherapy-related toxicities were analyzed weekly and registered as the higher score occurred during and 3 months after radiotherapy, according to the Common Toxicity Criteria for Adverse Effects (CTCAE) v.4.03.

The RT completion date was chosen as reference for measuring survival data. Relapse Free Survival (RFS) was measured as months free from local/distant relapse after the end of radio-chemotherapy; Overall Survival (OS) was the time from the end of radio-chemotherapy to death for any cause or last follow up, for living patients.

### Statistical analysis

The differences between the two treatments were investigated through the χ^2^ test.

OS and RFS were calculated through the Kaplan–Meier method and the differences evaluated with the Log-Rank Test.

The Propensity Score matched analysis (PS) (OS and RFS) was introduced to minimize the effect of confounding factors and to create two homogeneous populations (w-CDDP vs 3w-CDDP). The variables to match the patients (2:1) were age, disease stage and performance status (Karnofsky Performance Status, KPS). At the end, 160 patients were evaluable after the match (114 and 46 patients respectively in the 1w and 3w-CDDp group).

The multivariate analysis was done (OS and RFS) with Cox Regression model, both before and after PS, including all the variables used in the univariate one.

After PS the univariate and multivariate analysis were applied on the group of patients with oropharynx disease (113 patients). A separate analysis “HPV-status”-related, was not performed for the exiguity of the analysis.

The statistical analysis was made using the IBM® *SPSS* Statistics® v25.0; the *p* values were considered significant when *p* ≤ 0.05.

## Results

One hundred sixty-six patients were included in the analysis. Seventy-five percent (n = 125) were male, 140 (84%) aged < 70 years, 109 in good general conditions (KPS = 90–100, 66%). Almost 50% (n = 84) were tobacco smokers and had a current use of alcohol (n = 90). The patient’s features for the series are shown in Table 1.

**Table 1:** Patients features in relation with chemotherapy schedule

Characteristics of patients	1w-CDDP (N = 114)	3w CDDP (N = 52)	χ^2^	Entire series N (%)
Gender				
Male	92 (80.7%)	33 (63.5%)	0.0017	125 (75.3%)
Female	22 (19.3%)	19 (36.5%)		41 (24.7%)
Age				
< 70 years	90 (78.9%)	50 (96.2%)	0.005	140 (84.3%)
> 70 years	24 (21.1%)	2 (3.8%)		26 (15.7%)
Baseline KPS				
90–100	60 (52.6%)	49 (94.2%)	0.000	109 (65.7%)
70–80	52 (45.6%)	3 (5.8%)		55 (33.1%)
60	2 (1.8%)	0 (0%)		2 (1.2%)
Tobacco use				
Currently < 10 cigarettes/die	14 (12.3%)	4 (7.7%)	0.000	18 (10.8%)
Currently 10–20 cigarettes/die	27 (23.7%)	5 (9.6%)		32 (19.3%)
Currently > 20 cigarettes/die	31 (27.2%)	3 (5.8%)		34 (20.5%)
Stopped smoking > 5 years	22 (19.3%)	10 (19.2%)		32 (20.5%)
Never smoking	20 (17.5%)	30 (57.7%)		50 (30.1%)
Alcohol				
Currently	75 (65.8%)	15 (28.8%)	0.000	90 (54.2%)
Past	17 (14.9%)	1 (1.9%)		18 (10.8%)
Never	22 (19.3%)	29 (55.8%)		51 (30.7%)
ND	0 (0%)	7 (13.5%)		7 (4.2%)

*1w-CDDP* weekly Cisplatin, *3w-CDDP* three-weekly Cisplatin, *KPS* karnofsky performance status, *ND* not declared

The distribution of the clinical characteristics was not homogeneous in the two groups (Table 1). Patients treated with 1w-CDDP were significantly older (*p* = 0.005), in worse general conditions (*p* = 0.000) and more frequently actual smokers and alcohol consumers (*p* = 0.000).

Primary disease site was oropharynx in 119 patients (71.7%). In 129 cases, the disease was in stage IV (77.7%). Human Papilloma Virus status (HPV) was determined in 36.14% (60) of cases (Table 2).

**Table 2:** Disease characteristics in relation with chemotherapy schedule in relation to chemotherapy schedule

Disease characteristic	1w-CDDP (N.114)	3w-CDDP (N.52)	*p*	Entire series (%)
Histology			0.125	
Squamous	109 (95.6%)	52 (100%)		161 (97%)
Other histology	5 (4.4%)	0 (0%)		5 (3%)
Site of the disease			0.001	
Oropharynx	72 (63.2%)	47 (90.4%)		119 (71.7%)
Hypopharynx	23 (20.2%)	2 (3.8%)		25 (25.1%)
Larynx	19 (16.7%)	3 (5.8%)		22 (13.3%)
Staging T (TNM 7th Ed)			0.057	
T1–T2	52 (45.6%)	32 (61.3%)		84 (50.6%)
T3–T4	62 (54.4%)	20 (38.5%)		82 (49.4%)
Staging N (TNM 7th Ed)			0.024	
N0	16 (14%)	2 (3.8%)		18 (10.8%)
N1	17 (14.9%)	6 (11.5%)		23 (13.9%)
N2	80 (70.2%)	40 (76.9%)		120 (72.3%)
N3	1 (0.9%)	4 (7.7%)		5 (3%)
Stage (AJCC 7th Ed)			0.009	
II	5 (4.4%)	1 (1.9%)		6 (3.6%)
III	28 (24.6%)	3 (5.8%)		31 (18.7%)
IV	81 (71.1%)	48 (92.3%)		129 (77.7%)
HPV			0.000	
Positive	12 (10.5%)	32 (61.5%)		44 (26.5%)
Negative	11 (9.6%)	5 (9.6%)		16 (9.6%)
ND	91 (79.8%)	15 (28.8%)		106 (63.9%)

*HPV* human papilloma virus, *TNM* tumor, node, metastases, *AJCC* American Joint Committee on Cancer, *1w-CDDP* weekly Cisplatin, *3w-CDDP* three-weekly Cisplatin

The two treatment groups appear to be non-homogeneous, with a statistically significant prevalence of oropharyngeal tumors (90% vs 65%, *p* = 0.001) and N2-3 disease (84.7% vs 71%, *p* = 0.02) in 3w-CDDP group, and an excess of T3-4 disease (54% vs 38%; *p* = 0.05) among w-CDDP patients. A higher rate of HPV determination and positivity is also evident in 3w-CDDP group (*p* = 0.000) (Table 2).

One hundred fourteen patients were treated with w-CDDP (40 mg/m^2^) and 52 with 3w-CDDP (100 mg/m^2^). The CDDP/m^2^ doses was 200–250 mg in 25.4% in w-CDDP and 23.1% in 3w-CDDP; > 250 mg/ m^2^ in 2.6% and 50% respectively in w- and 3w-CDDP (*p* = 0.000). No patients had neo-adjuvant chemotherapy. CDDP was interrupted in 49.5% patients: 56.1% and 34.6% in w and 3w groups respectively (*p* = 0.012). CDDP was mostly interrupted in patients treated with dose/fraction > 2 Gy (59.6% vs 44%; *p* = 0.052) and in the w-CDDP.

All patients were treated with radical radiotherapy using different fractionations in relation to the clinical institutional use, assuming the same biological curative effect in combination with chemotherapy [15]: 10 (6%) patients had 69 Gy in 30 fractions (dose/fraction, 2.3 Gy/die); the others had 70 Gy in 2 Gy/fr (109 pts—66%) or 69.3 Gy with a slightly higher daily fractionation 2.1–2.12 Gy/fr (47 pts—28%). Dose/fraction > 2 Gy was mostly used in the weekly-CDDP group. Almost all patients had IMRT (Table 3).

**Table 3:** Treatment in relation to chemotherapy schedule

Treatment characteristic	1w-CDDP (N.114)	3w-CDDP (N.52)	*p*	Entire series (%)
Cumulative CDDP/m^2^ dose			0.000	
≤ 200 mg/m^2^	82 (71.9%)	14 (26.9%)		96 (57.8%)
200–250 mg/m^2^	29 (25.4%)	12 (23.1%)		41 (24.7%)
> 250 mg/m^2^	3 (2.6%)	26 (50%)		29 (17.5%)
Median CDDP/m^2^	175.9 mg/m^2^	248.1 mg/m^2^	0.026	
CDDP interruption				
Yes	64 (56.1%)	18 (34.6%)	0.012	82 (49.4%)
No	50 (43.9%)	34 (65.4%)		84 (50.6%)
RTT dose			0.000	
69 Gy	10 (8.8%)	0		10 (6%)
> 69 Gy and < 70 Gy	47 (41.2%)	0		47 (28%)
70 Gy	57 (50%)	52(100%)		109 (66%)
RTT dose/fraction			0.000	
2.3 Gy/fr	10 (8.8%)	0		10 (6%)
2.1–2.2 Gy/fr	47 (41.2%)	0		47 (28%)
2 Gy/fr	57 (50%)	52(100%)		109 (66%)
RTT technique			0.000	
3D	3 (2.6%)	4 (7.7%)		7 (4.2%)
IMRT (VMAT)	53 (46.5%)	48 (92.3%)		101 (60.8%)
Helical IMRT	58 (50.9%)	0 (0%)		58 (34.9%)

*1w-CDDP* weekly Cisplatin, *3w-CDDP* three-weekly Cisplatin, *RTT* radiotherapy, *IMRT* intensity modulated radiation therapy, *VMAT* volumetric modulated arch therapy, *fr* fraction

Two sub-analysis were conducted on groups of patients with homogeneous KPS. In the group with KPS < 90 (n = 57) no differences are evident between patients submitted to different chemotherapy schedules (age, smoking habits, site of disease). No differences are evident even in terms of interruption or dose of CDDP/m^2^.

On the other hand, in the group with KPS 90–100 (n = 109), the patients treated with different schedules are homogeneous only for age. A higher percentage of non-smokers (61.2% vs 16.7%; *p* = 0.000) and lower of alcohol users (28.6% vs 58.3%; *p* = 0.000) were treated with the 3w-CDDP schedule. Less patients treated with the 3-weekly schedule of this subgroup, interrupted chemotherapy (32% vs 50%; *p* = 0.081) and received < 200 mg/m^2^ of CDDP (24.5% vs 63.3%; *p* = 0.000).

### Acute toxicity

The rate of G3-4 acute hematological toxicity was 19.9% in the whole group (18.4% and 23.1% in 1w-CDDP and 3w-CDDP respectively (*p* = ns). G1-2 anemia and leucopenia were similar in the two groups; G1-2 thrombocytopenia was slightly more frequent in patients treated with w-CDDP (*p* = 0.01) (Table 4). Overall G3-4 mucositis, dermatitis and dysphagia rate were 33%, 10.8% and 19% respectively. G 3–4 emesis was higher in the group treated with w-CDDP (*p* = 0.007) while G1-2 acute xerostomia was more frequent in the group treated with 3w-CDDP (*p* = 0.009). No severe renal toxicity was recorded (Table 4).

**Table 4:** Acute toxicity as registered during the treatment

Acute toxicity	1 W-CDDP (N.114)	3 W-CDDP (N.52)	*χ*^*2*^	Entire series
Whole hematol tox			*0.285*	
G0	9 (7.9%)	1 (1.9%)		*10 (6%)*
G1–G2	84 (73.7%)	39 (75%)		*123 (74%)*
G3–G4	21 (18.4%)	12 (23.1%)		*33 (20%)*
Anemia			0.37	
G0	11 (9.6%)	6 (11.5%)		17 (10.2%)
G1–G2	102 (89.5%)	44 (84.6%)		146 (88%)
G3–G4	1 (0.9%)	2 (3.8%)		3 (1.8%)
Leucopenia			0.524	
G0	16 (14%)	7 (13.5%)		23 (14%)
G1–G2	80 (70.2%)	33 (63.5%)		113 (68%)
G3–G4	18 (15.8%)	12 (23.1%)		30 (18%)
Thrombocytopenia			0.01	
G0	26 (22.8%)	24 (46.2%)		50 (30%)
G1–G2	85 (74.6%)	27 (51.9%)		112 (67.5%)
G3–G4	3 (2.6%)	1 (1.9%)		4 (2.5%)
Kidney injury			0.111	
G0	89 (78.1%)	46 (88.5%)		135 (81.4%)
G1–G2	25 (21.9%)	6 (11.5%)		31 (18.6%)
G3–G4				
Mucositis			0.637	
G0	2 (1.8%)	–		2 (1.2%)
G1–G2	72 (63.2%)	36 (69.2%)		108 (65%)
G3–G4	39 (34.2%)	16 (30.8%)		55 (33.2%)
ND	1 (0.9%)	–		1 (0.6%)
Dermatitis			0.067	
G0	4 (3.5%)	–		4 (2.5%)
G1–G2	88 (77.2%)	48 (92.3%)		136 (81.9%)
G3–G4	14 (12.6%)	4 (7.7%)		18 (10.8%)
ND	8 (7%)	–		8 (4.8%)
Xerostomia			0.009	
G0	29 (25.4%)	9 (17.3%)		38 (22.9%)
G1–G2	68 (59.6%)	43 (82.7%)		111 (66.9%)
G3–G4	4 (3.5%)	–		4 (2.4%)
ND	13 (11.4%)	–		13 (7.8%)
Dysphagia			0.312	
G0	18 (15.8%)	5 (9.6%)		23 (13.8%)
G1–G2	72 (63.2%)	39 (75%)		111 (66.9%)
G3–G4	24 (21.1%)	8 (15.4%)		32 (19.3%)

*1w-CDDP* weekly Cisplatin, *3w-CDDP* three-weekly Cisplatin, *Nd* not declared

The rate of CDDP interruption was slightly higher (*p* = 0.052) in patients treated with higher fractional dose (44% and 56% in the 2 Gy/fr and > 2 Gy/fr, respectively); the same was true for cutaneous toxicity (8.3% and 15.8%, in the 2 Gyfr and > 2 Gy/fr, respectively). Patients with CDDP interruption had mostly G3-G4 vs G1-2 hematological toxicity (66.7% vs 33.3% *p* = 0.81).

### Overall survival

#### Univariate analysis before and after propensity score matched analysis

After a median follow-up of 32 months (respectively 35 and 26.5 m for the 1w and 3w-CDDP), the 1, 2 and 5 years actuarial OS of the entire series were 97%, 88% and 81.5%. Median OS was not reached neither in entire series nor in the two groups separately (1w and 3w-CDDP).

*Before Propensity scored analysis*, only female patients showed a statistically significant better OS compared with male patients (Table 5). OS was significantly better in patients with oropharyngeal disease as opposed with hypo-pharyngeal/laryngeal disease (*p* = 0.04): 1-year survival rates were 99.1%, 88%, 95.5%, and 2-and 5-year rates of 92.3%, 75.1%, 70.6% and of 89%, 59.1%, 73.3% for oropharyngeal, hypo-pharyngeal and laryngeal cancers, respectively.

**Table 5:** One- and two year overall (OS) survival before and after the propensity score matched analysis (PS)

Characteristic	OS univariate pre-PS			OS multivariate pre-PS		OS univarite after PS			OS multivariate after PS	
	1 year	2 year	*p*	Exp B	*p*	1 year	2 year		Exp B	
Gender			0.004		NS			0.022		0.034
Male	95	83.7				95.9	80.9		1	
Female	100	100				100	94.9		0.217	
Age			NS		NS			NS		NS
< 70 years	96.3	88.2				96.2	84.7			
> 70 years	100	85.9				100	81.2			
KPS			NS		NS			NS		NS
90–100	96.3	89.9				96.1	84			
70–80	98	83.4				98.1	83.7			
Tobacco use			NS		NS			NS		NS
Currently < 10 cig/die	94.1	94.1				94.1	94.1			
Currently 10–20 cig/die	96.8	90.1				96.7	89.7			
Currently > 20 cig/die	97.1	75.3				97.1	75.3			
Stopped > 5 years	96.6	88.2				96.6	84.7			
Never smoked	98	93				97.8	83.5			
Alcohol			NS		NS			NS		NS
Currently	95.4	85.3				95.3	85.1			
Past	100	82.4				100	82.4			
Never	98	93.1				97.9	77.9			
Stage T			NS		NS			NS		NS
T1-2	96.3	88.4				96	83.2			
T3-4	97.5	87.4				97.5	84.9			
Stage N			NS		NS			0.010		0.047
0	100	93.3				100	93.3		1	
1	95.7	91.3				95.7	91.3		0.780 (IC 0.09–6.773)	0.822
2	97.4	86.8				97.3	82.3		2.933 (IC 0.582–14.768)	0.192
3	80	80				60	60		14.936 (IC 1.665–133.985)	0.016
Stage of disease			NS		NS			NS		NS
II	100	100				100	100			
III	96.8	79.5				96.8	79.5			
IV	96.8	89.6				96.6	84.7			
Site of disease			0.04		0.027			0.034		0.007
Oropharynx	98.2	92.3		1		99.1	87		1	
Hypopharynx	88	75.1		6.238 (IC 1.549–25.4)	0.10	88	75.1		5.5 (IC 2.1.918–16.03)	0.002
Larynx	95.5	80.6		1.399 (IC 0.27–7.236	0.689	95.5	80.6		2.02 (IC 0.586–7.481)	0.255
RT technique			NS		ns			NS		ns
IMRT/VMAT	94.9	85.9				94.7	80			
Helical IMRT	100	89.7				100	89.7			
Type of CHT			0.026		0.007			NS		ns
1w CDDP	96.4	84.6		1		97.8	82.9			
3w CDDP	98	95.4		0.006 (0.000–0.241)		96.4	84.6			
Total CDDP/m^2^			NS		0.006			NS		ns
≤ 200 mg/m^2^	95.7	87.6		1		90	58.3			
> 200–250 mg/m^2^	100	88.4		0.567 (IC 0.123–2.627	0.469	100	78.6			
> 250 mg/m^2^	93.1	88.7		235.838 (IC 7.565–7352.1)	0.002	97	86.7			
CDDP interruption			NS		ns			NS		ns
Yes	97.5	87.6				97.3	85.7			
No	96.4	88.2				96.3	82.8			

*RTT* radiotherapy, *IMRT* intensity modulated radiation therapy, *VMAT* volumetric modulated arch therapy, *1w-CDDP* weekly Cisplatin, *3w-CDDP* three-weekly Cisplatin, *NS* not significant

OS is different in relation with 3 months nodal response: complete response, partial response and nodal progression are respectively linked with 1- and 2-year OS of 99%, 97%, 83% and 92%, 83%, 67%, respectively (*p* = 0.04).

Univariate analysis showed that OS was inferior with 1w-CDDP (*p* = 0.026); 12, 24- and 60-months survival rates were 96.4% versus 98%, 84.6% vs 95.4 and 75.9% versus 95.4, respectively in the 1w-CDDP versus 3w-CDDP. The different doses (CDDP/m^2^) did not impact significantly survival rates neither if used as categorical or continuous variables. Survival was better, without statistically significance, for patients who did not interrupt chemotherapy. The other clinical and therapeutic variables did not show statistically differences (Table 5).

*After propensity score matched analysis* the statistically significant better OS in female patients (*p* = 0.041) and in oropharyngeal disease (*p* = 0.047) was confirmed. The worse prognosis of patients with more extensive nodal involvement (N3, *p* = 0.011) was also demonstrated. Better OS for patients treated with 3w-CDDP was not confirmed (Table 5).

**Fig. 1:**
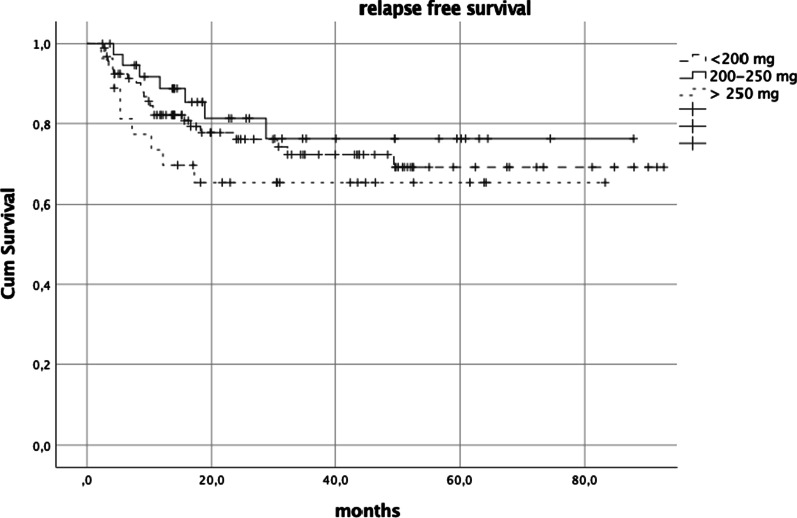
OS univariate analysis after PS in patients with oropharyngeal disease (*p* = 0.016)

In the group of patients with oropharyngeal disease, at univariate analysis after PS, the variable significantly influencing overall survival was total CDDP dose (*p* = 0.016) (Fig. 1).

#### Multivariate analysis before and after propensity score matched analysis

The multivariate analysis *before the propensity score analysis* showed better survival in patients with oropharyngeal cancer, treated with 3w-CDDP and with higher total CDDP/m^2^ (Table 5).

The analysis *after PS* demonstrated better survival in patients with oropharyngeal disease and low nodal disease burden. None of the therapeutic factors related to chemotherapy or radiotherapy, revealed impact on OS (Table 5).

Within the oropharyngeal disease group, the multivariate after PS confirmed that dose of CDDP maintained a slightly statistically significant impact on survival (*p* = 0.07) with lower death risk in patients treated with higher doses: ExpB 0.418 of 200–250 mg and 0.061 of > 250 mg in comparison to < 200 mg CDDP dose).

### Relapse free survival

#### Univariate analysis before and after propensity score matched analysis (PS)

Mean relapse free survival (RFS) was 69 months (range 63–75 months). Median RFS was not reached neither before nor after the propensity scored analysis.

At univariate analysis, *before PS*, RFS was not related to chemotherapy (1w-CDDP vs 3wCDDP) (*p* = 0.21) with 12- and 24-months survival rates of 85% versus 74%, 79% versus 67.5% in 1w-CDDP versus 3w-CDDP group, respectively. The other variables did not show statistically significant differences.

**Fig. 2:**
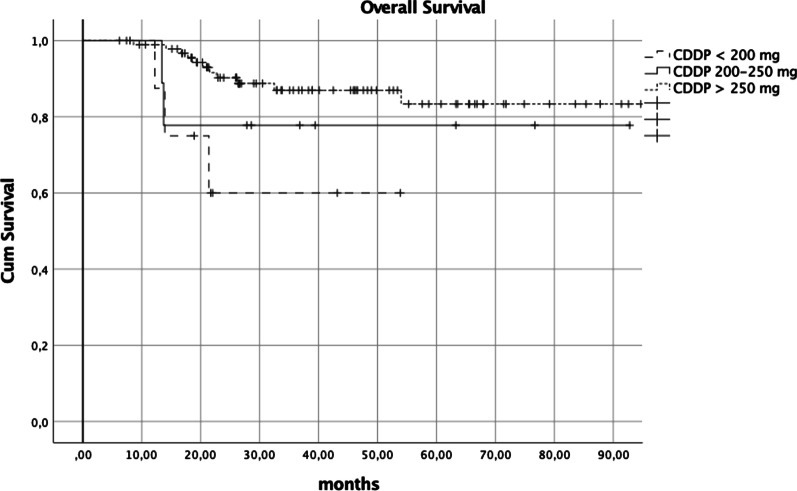
RFS univariate analysis after PS (*p* = not significant)

The results *after the propensity score match* were almost the same as those registered before applying the propensity analysis (Table 6) (Fig. 2).

**Table 6:** Relapse free survival before and after the propensity score matched analysis (PS)

Characteristic	RFS univariate pre PS			RFS multivariate pre-PS		RFS univariate post PS			RFS multivariate post-PS	
	1 year	2 year	*p*	Exp B	*p*	1 year	2 year	*p*	Exp-B	*p*
Gender			NS		0.035			0.027		0.015
Male	78.7	71				78.7	71		1	
Female	90.2	86.8				94.2	90.3		0.229 (IC 0.07–0.7488)	
Age			NS		NS			NS		NS
< 70 years	80.6	75				81.2	74.1			
> 70 years	87.7	82.2				87.7	82.2			
KPS			NS		NS			NS		NS
90–10	80.9	74.9				81.7	75.4			
70–80	82.5	74.8				82.5	74.8			
Tobacco use			NS		NS			NS		NS
Currently < 10 cigarettes/die	83	75.4				83	75.4			
Currently 10–20 cigarettes/die	87	77.6				86.5	76.6			
Currently > 20 cigarettes/die	78.3	73.8				78.3	73.7			
Stopped smoking > 5 years	82.5	78.7				81.8	77.9			
Never smoked	79.5	72.9				82.3	74.9			
Alcohol			NS		NS			NS		NS
Currently	85.8	79				85.7	78.7			
Past	76.7	63.8				76.7	63.8			
Never	78.4	76.2				78.7	76.3			
Stage T			NS		NS			NS		NS
T1-2	82.5	77.9				83.6	78.9			
T3-4	80.9	72.1				80.9	72.1			
Stage N			NS		NS			NS		NS
0	88.9	82.1				88.9	82.1			
1	91.1	85				91.1	85			
2	79.8	74.2				80.4	74.5			
3	53.3	26.7				26.7	26.7			
Stage of disease			NS		NS			NS		NS
II	100	100				100	100			
III	83.4	79.5				83.4	79.5			
IV	80.4	72.8				81	73.1			
Site of disease			NS		NS			NS		NS
Oropharynx	84.9	78.8				85.9	79.4			
Hypopharynx	84	68.5				84	68.5			
Larynx	63	63				63	63			
RT technique			NS		NS			NS		NS
IMRT/VMAT	76.7	69.5				77.5	70			
Helical IMRT	92.3	82.6				92.3	86.4			
Type of CHT			NS		NS			NS		NS
1w-CDDP	85.2	78.9				75.2	67.6			
3w-CDDP	74.3	67.5				85.2	78.9			
Total CDDP/m^2^			NS		NS			NS		NS
< 200 mg/m^2^	66.7	53.3				48	48			
200–250 mg/m^2^	85.9	85.9				93.3	93.3			
> 250 mg/m^2^	82.3	75.6				82.9	76			
CDDP interruption			NS		NS			NS		NS
Yes	79.6	76.2				79.8	76.3			
No	83.8	74.2				84.7	74.7			

*RT* radiotherapy, *IMRT* intensity modulated radiation therapy, *CDDP* cisplatin, *1w-CDDP* weekly cisplatin, *3w-CDDP* three weekly cisplatin

The same results were confirmed, both to univariate and multivariate analysis, within the group of patients with oropharyngeal disease (data not shown).

The loco-regional (T and N) median free survival was not statistically different between the two groups neither before (*p* = 0.453) nor after (*p* = 0.394) propensity score analysis.

#### Multivariate analysis after PS

The multivariate analysis confirmed the gender as independent factor predicting RFS (Table 6).

## Discussion

Due to its ability to increase the tumoricidal activity of radiotherapy, cisplatin is the standard agent, in combination with radiotherapy, to treat LAHNSCC fit patients, both with curative and postoperative intent [2, 4, 16–18].

Although several papers about the use of different CDDP schedules are present, the 3w-CDDP regimen, supported by level 1 data, show a significant increase in overall survival and loco-regional disease control compared to radiotherapy alone [1–4, 19]. Despite benefit in terms of disease control, this chemotherapy schedule is burdened by severe toxicity, both acute and chronic, in particular myelotoxicity and mucositis [6].

Adequate pretreatment patients’ characteristics remain crucial and difficult to be determined upfront. Indeed, frail patients (with older age and low performance status) could benefit from less toxic chemotherapy regimens [20–22].

Many efforts have been made to identify an alternative CDDP schedule achieving optimal disease control with minimal complications in order to reduce toxicity and, possibly, treatment interruptions that could compromise the treatment efficacy.

Different meta-analyses have been published on the topic. Jian [5] analyzed studies published from 2006 to 2014 comparing weekly Cisplatin (25–40 mg/m^2^) with the three-weekly one (Cisplatin at 80–100 mg/ m^2^), in combination with radiotherapy for the treatment of stage II-IV head and neck cancers (including nasopharynx). No significant differences in 2-(Hazard Ratio—HR—1.05, *p* = 0.85) and 3-year OS (HR 1.12, *p* = 0.65) were evident between the two schedules; also, 1- and 2-year Local Relapse Free Survival (LRFS) were similar, (HR 1.26, *p* = 0.65 and 1.14, *p* = 0.74 respectively). Better 5-year OS (HR 1.75, *p* = 0.006) was registered for the 3w-CDDP schedule. In this paper, however, it is not clearly defined if patients treated with 3w-CDDP had a better KPS or if KPS influences the outcome. The reported better long-term survival, evaluated only on two included papers, could thus be related to this important clinical aspect. In terms of acute toxicity, the two groups showed the same hematological toxicity (leukopenia, anemia, thrombocytopenia); less frequent severe intestinal toxicities (nausea and vomiting) were registered in the 1w-CDDP group (*p* = 0.006), whereas severe mucosal toxicity and CDDP delay/interruption were more common in patients with non-nasopharyngeal cancer in the 1-CDDP group (*p* < 0.0001). As far as treatment compliance is concerned, the data are very heterogeneous, since a significant proportion of patients (42% in the weekly CDDP group vs 30% in the three-weekly group) received neo-adjuvant chemotherapy, possibly reducing the tolerance to the concomitant phase. Another limitation of this study is the cumulative analysis of very different disease sites (including nasopharynx) and of different w-CDDP doses (range, 25–40 mg/m^2^/w).

Carlsson et al. [23] al retrieved from literature review 13 studies (prospective and retrospective, published between 2014 and 2016) on definitive chemoradiation with single-agent CDDP administered with three different schedules (3w, 1w/daily). Estimated 3-years OS was 68% and 61% for 3w versus 1w/daily regimens, respectively. Similar results were obtained by Jacinto et al. who analyzed seven studies including both primary and adjuvant treatments. No differences were found in terms of 1-year OS. Moreover, data pooled form six of the selected studies showed similar results between the two arms for clinical outcome (5-year PFS) and toxicity profile (renal events, mucositis, dermatitis, treatment interruption and number of patients receiving at least 200 mg/m^2^ CDDP). Szturz et al. [7] performed a more extensive analysis (52 studies), comparing adjuvant/radical 1w-CDDP and 3w-CDDP concomitant to radiotherapy. Results did not show a statistically significant difference in OS and relapse rate between the two treatments. Three-weekly administration, however, appeared to be linked with more severe myelosuppression (leukopenia, *p* = 0.0083 and thrombocytopenia, *p* = 0.0024), gastrointestinal toxicity (*p* < 0.001) and severe nephrotoxicity (*p* = 0.0099), while there were no significant differences in mucosal toxicity. Three-weekly administration was also related to inferior compliance: only 71% of patients completed the full chemotherapy treatment as compared to 88% of the patients who had w-CDDP. It is also worth noting the different distribution of the disease sites in the two groups, with a higher prevalence of oropharynx cancer in the group undergoing three-weekly chemotherapy (49% vs 36%). A more recent meta-analysis focused on the comparison between 3-w and 1w CDDP, including only randomized controlled trial [12]. Results based on 6 studies again confirmed that low dose 1-w CDDP was not associated neither with improved oncologic outcomes nor with lower acute toxicity. Heterogeneity data among the analyzed clinical trials (in terms of both chemotherapy regimens and radiation therapy techniques) as well as the lack of long-term toxicity data represented the main weaknesses of the study.

Recently, along with the reported meta-analyses, phase III randomized trials also have been published. Noronha et al. [6] designed a non-inferiority study, investigating the outcome of patients with LA head-neck carcinoma (except nasopharynx) treated with 30 mg/m^2^ w-CDDP compared to the 3w-CDDP 100 mg/m^2^ in postoperative/radical setting. The main endpoint of the study was loco-regional control; the secondary ones included toxicity, compliance and OS. The study included 300 patients (150/arm) but 93% were in a postoperative setting (87.3% oral cavity tumors). The 2-year loco-regional control was significantly higher for the 3w-CDDP (*p* = 0.014). The results were confirmed after the comparison of patients receiving total CDDP dose > 200 mg/m^2^. As for Progression Free Survival (PFS) and OS, however, no statistically significant differences were registered. Regarding toxicity, the 3w regimen was burdened by more frequent severe acute toxicity (*p* = 0.006) and the hospitalization rate was greater (*p* < 0.001). The main limitation of this study is the small rate of patients treated with radiotherapy alone, due to the preponderance of oral cavity tumors, and the low dose of Cisplatin administered in the weekly schedule (30 mg/m^2^), compared to the standard of 40 mg/m^2^. Results of a recent randomized trial performed on 77 patients (39 weekly and 38 3-W CDDP) head and neck cancer patients focused on chemotherapy-related toxicity have been presented by Ameri et al. [8]. Low dose CDDP was administered at 40 mg/m^2^ and renal indices were considered along with hematologic toxicity. The average estimated glomerular filtration rate (eGFR) resulted to be significantly higher in the 3w cohort. Moreover, treatment interruption resulted to be primarily due to neutropenia in the 3w group while renal failure and thrombocytopenia were more frequent among patients treated with the weekly schedule.

Considering the patient reported outcomes, Arbab et al. [9] performed a retrospective analysis on 99 patients (73 patients treated with a 1w schedule and 26 with a 3w). Results showed that patients reported outcome resulted to be comparable among the two cohorts of patients.

There have also been several attempts to substitute chemotherapy with cetuximab in old and bad general conditions patients, although the Bonner’s Study wasn’t designed for such patients [24, 25]. The results of these studies are not uniform, but the data of the De-Escalate and RTOG 1016 prospective trials [26, 27] as well as those of a smaller Italian trial [28, 29] with an emphasis on toxicity, did not confirm the hypothesis of the better compliance and equal efficacy of bio-radiotherapy, particularly in patients with better prognosis (HPV positive disease).

In this context, our study aims to contribute to the body of literature on this controversial issue with a retrospective evaluation of the efficacy and tolerability of the two chemotherapy schedules (1w-CDDP 40 mg/m^2^ and 3w-CDDP 100 mg/m^2^) administered concurrently with radiotherapy in patients with LA head-neck cancer (oropharynx, hypopharynx and larynx).

The two treatment groups, in our series, are significantly different in relation to patient clinical characteristics (per arm number of patients, gender, age, performance status, alcohol, and smoking habits); higher rates of women, young patients and subjects with better KPS and less smoking and alcohol consumption were registered in the 3w-CDDP group. Moreover, in the same group there was a prevalence of oropharynx cancer, even if they had more advanced nodal disease. Nevertheless, the propensity score method applied for the statistical analysis was able to mitigate these inhomogeneities thus rendering more reliable and robust the presented results.

A non-significantly higher rate of G3-4 hematologic toxicity was observed for the 3-weekly schedule. No significant differences were evident in terms of mucositis or dysphagia. A higher rate of G1-2 thrombocytopenia, mild gastrointestinal toxicity and CDDP interruptions were observed in patients treated with w-CDDP. The higher rate of toxicities could be attributed to the different characteristics of patients treated: more patients with low KPS, older than 70 years and smoke and alcohol addiction were treated with w-CDDP. The subgroup analysis showed that also within subgroup with KPS ≥ 90 patients of the 1w-CDDP group, are more frequently alcohol and smoking user.

The OS analysis of the present series, not corrected for age, performance status and disease stage, showed a statistically significant better survival for patients treated with 3w-CDDP compared to w-CDDP, with 2- and 5-years rates of 95.4% versus 84.6% and 95.4% versus 75.9%, respectively (*p* = 0.026). This result is, probably, related with a selection bias of the patients in the 3w-CDDP group (younger age, better performance status, less smoking and alcohol consumption, and higher rate of HPV positivity). This interpretation of the data is confirmed by the similar survival results registered in the two treatment groups with the propensity scored matched analysis.

The same results have been obtained also for RFS. Similar results (no differences in terms of 5-year OS and cancer-specific survival) were obtained by Han et al. [13] in a matched pair analysis on 472 pts (283 treated with 3-w CDDP and 189 treated with 30–50 mg/m^2^ once weekly). Attention should be posed to give the higher CDDP dose, both with 1w- or 3w schedule, for patients with oropharyngeal disease. Moreover, results from the current multivariate analysis after the propensity scored matched analysis on both for OS and RFS showed that neither the interruption of chemotherapy nor the CDDP total dose/m^2^ can be identified as an independent prognostic factor.

Propensity score analysis is useful to decrease the biases related to the analysis of a non-randomized population, that however cannot be completely eliminated.

## Conclusions

Three-weekly CDDP still represents the gold standard in curative and postoperative concurrent chemoradiation for LAHNSCC patients, despite the definition of the gold standard of the chemotherapy schedule is still much debated.

This is a retrospective—propensity score matched—analysis suggesting the two CDDP schedules are not different in terms of survival outcomes. However, these data, since they are retrospective in nature, are not per se sufficient to modify current clinical practice but could confirm, together with other already published data, that 1w-CDDP can be safely used in this group of patients. The lower patients’ compliance to the 1w-CDDP schedule could be justified by the worst patients’ prognostic factors (older age and lower performance status, alcohol consumption and smoking habits) compared to the 3w-CDDP cohort but it should be taken into account when we choose this personalized approach to support the frailty.

## Data Availability

The datasets analysed during the current study are available from the corresponding author on reasonable request.
